# Supplementary material for: MetaRibo-Seq measures translation in microbiomes
Source: Nat Commun. 2020 Jun 29;11:3268. doi: 10.1038/s41467-020-17081-z (PMC7324362; doi:10.1038/s41467-020-17081-z)
Supplement: Supplementary file 10 — Supplementary Data 7 [file 41467_2020_17081_MOESM10_ESM.zip › File2/Confidence_High_Taxonomy/442204_out.krona.html]

Javascript must be enabled to view this page.

members
magnitude
magnitudeUnassigned
count
unassigned
taxon
rank

442204\_out

200

200
superkingdom
2

200
phylum
976

200643
200
class

171549
200
order

family
2
2005525

2
genus
375288

46503
2
species

SRS017247\_contig\_number\_2702SRS098644\_contig\_number\_13595

family
1
171552

577309
genus
1


SRS013521\_contig\_number\_4568
1
species
454154

family
197
815


SRS050422\_contig\_number\_21311
816
genus
197
1


SRS024075\_contig\_number\_863
818
1
species


SRS043667\_contig\_number\_4692
585544
1
species


SRS145497\_contig\_number\_3581
1262737
1
species

species
93
28116

SRS011084\_contig\_number\_21566SRS012902\_contig\_number\_1292SRS013215\_contig\_number\_613SRS013476\_contig\_number\_2896SRS013521\_contig\_number\_4474SRS014235\_contig\_number\_42562SRS014287\_contig\_number\_8801SRS014459\_contig\_number\_19825SRS014613\_contig\_number\_15063SRS014683\_contig\_number\_1697SRS014979\_contig\_number\_36740SRS015065\_contig\_number\_26074SRS015431\_contig\_number\_7247SRS015794\_contig\_number\_1886SRS016018\_contig\_number\_8839SRS016840\_contig\_number\_2774SRS017191\_contig\_number\_14017SRS017745\_contig\_number\_1907SRS018313\_contig\_number\_10800SRS018817\_contig\_number\_24412SRS019068\_contig\_number\_76204SRS019445\_contig\_number\_29696SRS019693\_contig\_number\_7008SRS019968\_contig\_number\_551SRS022071\_contig\_number\_14662SRS023346\_contig\_number\_1865SRS023829\_contig\_number\_35SRS024132\_contig\_number\_3278SRS024549\_contig\_number\_4171SRS024663\_contig\_number\_13883SRS045244\_contig\_number\_1589SRS045739\_contig\_number\_10395SRS046369\_contig\_number\_13829SRS046712\_contig\_number\_2053SRS049896\_contig\_number\_23700SRS049959\_contig\_number\_29594SRS050299\_contig\_number\_9629SRS050801\_contig\_number\_4057SRS051031\_contig\_number\_28989SRS053214\_contig\_number\_6403SRS053398\_contig\_number\_20636SRS053573\_contig\_number\_683SRS054352\_contig\_number\_15417SRS058268\_contig\_number\_419SRS062427\_contig\_number\_1716SRS064232\_contig\_number\_3367SRS064276\_contig\_number\_15086SRS074670\_contig\_number\_4532SRS074964\_contig\_number\_410SRS075398\_contig\_number\_1146SRS076976\_contig\_number\_15733SRS077194\_contig\_number\_24066SRS077454\_contig\_number\_1631SRS077502\_contig\_number\_15563SRS078242\_contig\_number\_20293SRS078665\_contig\_number\_4308SRS097958\_contig\_number\_24132SRS098061\_contig\_number\_13740SRS098655\_contig\_number\_13223SRS098881\_contig\_number\_21197SRS104084\_contig\_number\_9697SRS1041037\_contig\_number\_contig-100\_27.94116SRS1041095\_contig\_number\_4007SRS1041118\_contig\_number\_16444SRS1041136\_contig\_number\_4798SRS1041137\_contig\_number\_30658SRS1041147\_contig\_number\_1477SRS104311\_contig\_number\_38385SRS104400\_contig\_number\_3592SRS104975\_contig\_number\_18101SRS1055034\_contig\_number\_6509SRS1055056\_contig\_number\_13313SRS1055095\_contig\_number\_2102SRS143372\_contig\_number\_2068SRS143417\_contig\_number\_5870SRS143466\_contig\_number\_8052SRS144183\_contig\_number\_32237SRS147039\_contig\_number\_2268SRS147088\_contig\_number\_contig-100\_691.52517SRS147425\_contig\_number\_10925SRS147653\_contig\_number\_5830SRS148159\_contig\_number\_42243SRS148319\_contig\_number\_3394SRS148721\_contig\_number\_46916SRS149075\_contig\_number\_10924SRS149181\_contig\_number\_20732SRS893253\_contig\_number\_897SRS893256\_contig\_number\_1266SRS893327\_contig\_number\_11291SRS893342\_contig\_number\_523SRS893369\_contig\_number\_8489SRS893378\_contig\_number\_13450SRS893382\_contig\_number\_2546

457387
species
4

SRS016495\_contig\_number\_18410SRS053335\_contig\_number\_17732SRS104197\_contig\_number\_contig-100\_131.3181SRS147766\_contig\_number\_6431

species
41
371601

SRS013158\_contig\_number\_426SRS014534\_contig\_number\_785SRS016335\_contig\_number\_29351SRS016897\_contig\_number\_1803SRS017521\_contig\_number\_23733SRS018049\_contig\_number\_1208SRS018888\_contig\_number\_3236SRS019286\_contig\_number\_5249SRS019787\_contig\_number\_13004SRS020271\_contig\_number\_4547SRS020869\_contig\_number\_10797SRS023971\_contig\_number\_663SRS043001\_contig\_number\_8705SRS043841\_contig\_number\_9852SRS044535\_contig\_number\_14509SRS045195\_contig\_number\_3365SRS047014\_contig\_number\_29150SRS048164\_contig\_number\_33237SRS049446\_contig\_number\_15134SRS064973\_contig\_number\_1276SRS075716\_contig\_number\_2782SRS075878\_contig\_number\_3440SRS077589\_contig\_number\_7709SRS101268\_contig\_number\_1582SRS1041036\_contig\_number\_1055SRS1041090\_contig\_number\_5966SRS1041130\_contig\_number\_4128SRS1041144\_contig\_number\_6568SRS1041145\_contig\_number\_27508SRS1041146\_contig\_number\_48SRS104636\_contig\_number\_18169SRS104912\_contig\_number\_contig-100\_200.108302SRS1055099\_contig\_number\_1690SRS140492\_contig\_number\_6134SRS142781\_contig\_number\_580SRS143181\_contig\_number\_11394SRS144362\_contig\_number\_13322SRS144506\_contig\_number\_48854SRS149325\_contig\_number\_5652SRS149879\_contig\_number\_27450SRS893380\_contig\_number\_462


SRS057717\_contig\_number\_996
457390
1
species


SRS018936\_contig\_number\_10311
species
1
1946086


SRS017622\_contig\_number\_28
2292956
species
1


SRS019496\_contig\_number\_16883SRS142599\_contig\_number\_6407
2
species
457389


SRS019397\_contig\_number\_3488SRS143876\_contig\_number\_186
species
2
46506


SRS016954\_contig\_number\_16489SRS051610\_contig\_number\_894
821
species
2


SRS011134\_contig\_number\_20342SRS013800\_contig\_number\_140SRS015782\_contig\_number\_3413SRS015794\_contig\_number\_contig-100\_1312.83411SRS016629\_contig\_number\_1975SRS018133\_contig\_number\_958SRS019445\_contig\_number\_30847SRS019496\_contig\_number\_17944SRS019685\_contig\_number\_33839SRS019685\_contig\_number\_40339SRS021153\_contig\_number\_406SRS023583\_contig\_number\_1268SRS024435\_contig\_number\_32004SRS042284\_contig\_number\_5220SRS044535\_contig\_number\_13902SRS047044\_contig\_number\_16814SRS049995\_contig\_number\_30031SRS050974\_contig\_number\_412SRS063040\_contig\_number\_29046SRS064645\_contig\_number\_1526SRS075821\_contig\_number\_22727SRS098571\_contig\_number\_40077SRS104693\_contig\_number\_11971SRS104975\_contig\_number\_24424SRS105082\_contig\_number\_11658SRS1054716\_contig\_number\_14747SRS142890\_contig\_number\_24957SRS143070\_contig\_number\_10239SRS146764\_contig\_number\_14669SRS147088\_contig\_number\_contig-100\_610.52436SRS147271\_contig\_number\_11553SRS147346\_contig\_number\_43440SRS149181\_contig\_number\_26726SRS893230\_contig\_number\_4267SRS893270\_contig\_number\_8236SRS893292\_contig\_number\_3107SRS893300\_contig\_number\_11621
37
species
338188


SRS043701\_contig\_number\_14508SRS050941\_contig\_number\_11374SRS077730\_contig\_number\_3321
817
species
3

1263045
1
species

SRS013638\_contig\_number\_15227


SRS052576\_contig\_number\_223SRS147919\_contig\_number\_778
2
species
1739517

3
species
47678

SRS016203\_contig\_number\_8810SRS098571\_contig\_number\_60096SRS142890\_contig\_number\_1767
